# Supplementary figures and images for: A non-canonical role for p27Kip1 in restricting proliferation of corneal endothelial cells during development
Source: PLoS One. 2020 Jan 13;15(1):e0226725. doi: 10.1371/journal.pone.0226725 (PMC6957298; doi:10.1371/journal.pone.0226725)

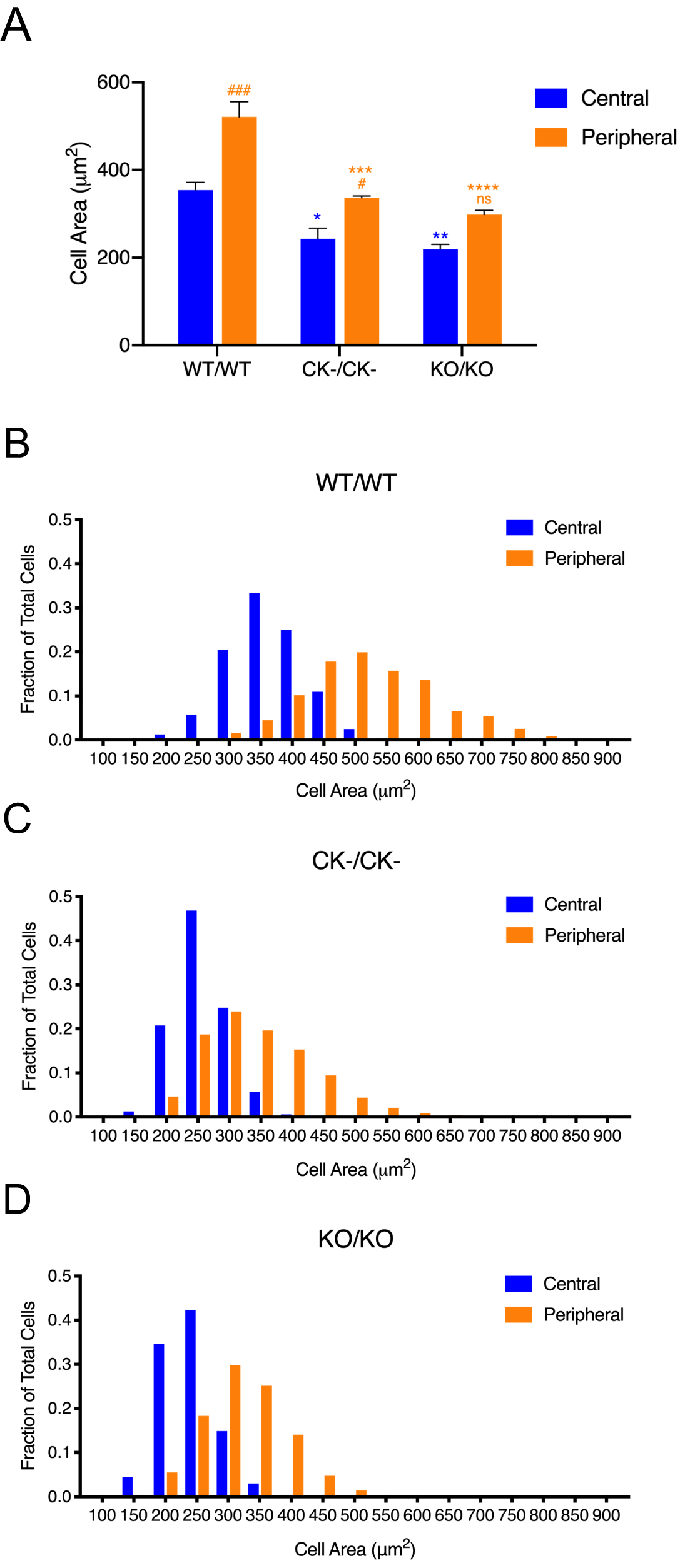

Supplement: S1 Fig — (A) Averaged data. Declines in individual cell area are evident for central and peripheral regions of both mutants, relative to the wild-type control. (B-D) Histograms plotting cell area distributions. In p27+/+ monolayers, the size ranges of regional populations are relatively broad and partially overlapping. Expression of mutant alleles leads to shifts in cell sizes to smaller values in both central and peripheral regions, as well as more compressed distributions and greater overlap. Data in (A) represent means ± SEM (n = 3). Ordinary two-way ANOVA followed by Tukey’s HSD test was performed. * and ** indicate p<0.05 and p<0.005 by comparison to wild-type (central), while *** and **** indicate p<0.0005 and p<0.0001 by comparison to wild-type (peripheral). # and ### indicate p<0.05 and p<0.001 by comparison to central regions. ns indicates not significant. (TIF) [file pone.0226725.s001.tif]

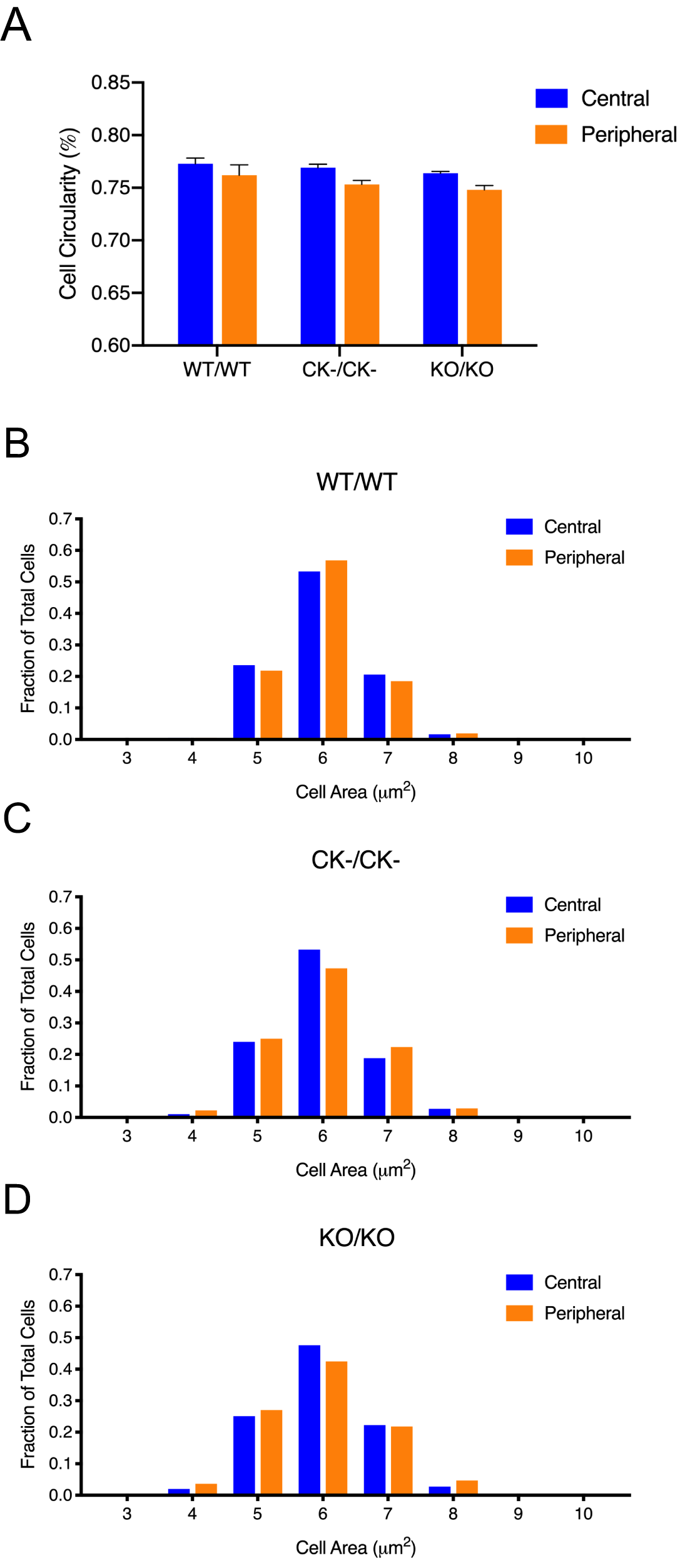

Supplement: S2 Fig — (A) Averaged circularity data. Peripheral cells exhibit a small, but significant, decline in circularity across all genotypes (p<0.01). However, no difference is observed in either of the two regional cell populations when comparing wild-type and mutant monolayers. (B-D) Histogram plots of nearest neighbor distributions. Quantitatively similar numbers of neighbors are seen for all genotypes. Data in (A) represent means ± SEM (n = 3). Ordinary two-way ANOVA followed by Tukey’s HSD test was performed. (TIF) [file pone.0226725.s002.tif]
